# Polyethylene Recovery from Multilayer Plastic Packaging Waste

**DOI:** 10.3390/polym18050638

**Published:** 2026-03-05

**Authors:** Anareth Cavuquila, Luanna Maia, Germano A. Carreira, Inês Portugal, Carlos M. Silva, Ana Barros-Timmons

**Affiliations:** 1 CICECO—Aveiro Institute of Materials, Department of Chemistry, University of Aveiro, 3810-193 Aveiro, Portugal; 2 LEPABE, ALiCE, Faculty of Engineering, University of Porto, Rua Dr. Roberto Frias, 4200-465 Porto, Portugal; 3 CERES, Department of Chemical Engineering, Faculty of Sciences and Technology, University of Coimbra, Rua Sílvio Lima, Polo II, 3030-790 Coimbra, Portugal; 4 i9Green, Av. D. José Alves Correia da Silva, Edifício Vela Sul 2° D, Rotunda Sul, Cova da Iria, 2495-402 Fátima, Portugal

**Keywords:** design of experiments, dissolution–precipitation, plastic recycling, response surface methodology

## Abstract

Multilayer plastic packaging waste (MPPW) represents a major challenge for waste management due to its widespread use in single-use applications and its complex, heterogeneous structure. Variations in polymer composition, layer thickness and number of layers significantly hinder conventional recycling processes, leading most MPPW to be disposed of through landfilling or incineration. This study presents the development and optimization of a dissolution–precipitation process using toluene to recover polyethylene (PE) from MPPW. The proposed method successfully produced PE with less than 5 wt% polypropylene (PP), meeting common recycling quality requirements. Design of experiments (DoEs) combined with response surface methodology (RSM) was applied to evaluate the influence of key operating parameters, including temperature, dissolution time, solvent to waste ratio and agitation speed, to identify optimal processing conditions. The results demonstrated that temperature had the most significant influence on both dissolution yield and polymer purity. Optimal conditions of 100 °C, 30 min, 400 rpm, and a solvent-to-waste ratio of 15 mL/g resulted in a total recovery yield of 39.1% with a polymer composition of 97.7 wt% PE and 2.3 wt% PP. Owing to the use of established and scalable unit operations, the process shows strong potential for industrial-scale implementation without requiring complex or specialized infrastructure.

## 1. Introduction

Recognizing the urgency of improving waste management, the European Union has committed to ensuring that all packaging placed on the EU market must be recyclable in an economically viable way by 2030 [1]. However, progress has been uneven across member states, with several countries struggling to meet these objectives [2]. This disparity highlights the need for improved recycling technologies and infrastructure to support effective waste management practices.

A significant part of this challenge lies in managing multilayer plastic packaging (MPP) [3], which is widely used in the food, pharmaceutical, and consumer goods industries due to its excellent barrier properties, mechanical strength, and ability to preserve products [4,5]. These benefits, however, come at an environmental cost because MPP is composed of different polymers, often combined with materials such as aluminum or paper [6]. Managing multilayer plastic packaging waste (MPPW) involves significant logistical and technical challenges, including high costs associated with sorting, complex waste streams prone to cross-contamination, and the inefficiency of existing recycling technologies [7,8]. These challenges are further exacerbated by the design of MPP, which prioritizes performance over recyclability and results in tightly bonded, heterogeneous materials that are extremely difficult to process using conventional mechanical recycling. Consequently, most MPPW ends up in landfills or incinerators [7,8].

Both industry and academia have been actively researching alternative technologies for dealing with MPPW, including delamination strategies to separate polymer layers [9,10,11], dissolution–precipitation of selected polymer layers [12,13,14], chemical depolymerization for monomer recovery [15,16], compatibilization to yield blends [17,18], and thermal degradation to recover hydrocarbons and energy [19,20]. Delamination involves separating the layers of MPPW using techniques such as chemical depolymerization of interlayers and/or the dissolution of adhesive layers. For instance, Ügdüler et al. [11] successfully delaminated five MPP films composed of polyolefin/aluminum/poly(ethylene terephthalate) (PO/Al/PET) with polyurethane-based adhesives using a 50–100 vol% formic acid aqueous solution at temperatures from 50 °C to 75 °C, achieving optimal results at 75 °C and 100 vol%. Additionally, Ügdüler et al. [16] explored alkaline hydrolysis for the depolymerization of PET, promoting the delamination of multilayer PET trays at temperatures up to 80 °C and atmospheric pressure. They found that multilayer structures reduced the depolymerization yield by half compared to monolayer PET, due to diffusion limitations. Aymonier and Slostowski [9] used supercritical carbon dioxide as a swelling agent combined with cosolvents, such as acetone and water, to dissolve adhesive layers and delaminate multilayer products, including food and pharmaceutical packaging, and shoes. High pressures (up to 500 bar) and moderate temperatures (60–130 °C) were recommended to avoid polymer degradation, with cosolvents tuning the selectivity for specific adhesives.

The selective dissolution–precipitation has emerged as a promising approach for addressing MPPW [21]. Conventional organic solvents, such as toluene, xylene, tetrahydrofuran (THF), dimethyl sulfoxide (DMSO), and N-methylpyrrolidone (NMP), and alternative solvents, such as biobased limonene and gamma-Valerolactone, ionic liquids and deep eutectic solvents, have been tested for the dissolution of polymers [22]. A notable example is the solvent-targeted recovery and precipitation (STRAP) process developed by Walker et al. [14], which uses solvents selected with the COSMO-RS predictive tool to dissolve MPP films. In this process, MPP films composed of polyethylene (PE), poly(ethylene-*co*-vinyl alcohol) (EVOH), and PET are recycled using solvent/antisolvent pairs, such as toluene/acetone for PE recovery and DMSO/water for EVOH recovery, obtaining PET as an undissolved film with a near 100% yield.

With respect to compatibilization, compatibilizers are used to produce polymer blends with suitable properties for new products. For instance, in the case of PE/PET blends, polyethylene-graft-maleic anhydride (PE-g-MA) and ethylene-glycidyl methacrylate (E-GMA) [23], as well as ethylene-propylene rubber (EPR) [18], have been studied as compatibilizers.

Pyrolysis has been used industrially for feedstock recycling of plastic waste, including MPPW. An example of the industrial application of this mature technology is the LyondellBasell MoReTec^®^ molecular recycling technology, which decomposes plastic materials from various origins, including MPPW, yielding pyrolysis oil, which is then processed to generate propylene and ethylene monomers for producing new plastic materials [24].

Each of the proposed technologies presents distinct advantages and limitations. Compatibilization can directly process mixed plastics; however, contamination levels and the need for multiple compatibilizers, when applied to blends of multiple polymers, limit its application. Selective dissolution–precipitation produces high-quality outputs but involves high solvent costs and potential hazards. The complexity of multilayer structures hinders chemical depolymerization. Thermal depolymerization can handle large volumes of waste but primarily results in downcycling, requiring additional processing with significant energy and material consumption to reproduce plastics [25,26]. These considerations underscore the need for ongoing research and development to improve the efficiency and feasibility of these technologies across various plastic waste streams.

The dissolution of a polymer into a solvent is governed by diffusion, polymer chain disentanglement, and chemical affinity. The solvent diffuses into the polymer, forming a gel-like swollen layer with two interfaces: one between the glassy polymer and the gel layer, and the other between the gel layer and the solvent, which over time leads to the dissolution of the polymer [27]. The critical parameters influencing dissolution efficiency are the solvent and polymer type, molar mass, solvent-to-polymer ratio, temperature, particle size, agitation, and time [27,28,29,30,31,32,33,34,35]. The solvent type is crucial for the target polymer’s effective and selective dissolution. This topic has been addressed by Achillias et al. [33] and Ferreira et al. [35], focusing on Hildebrand and Hansen solubility parameters, and Walker et al. [14] using thermodynamic predictions with COSMO-RS.

The design of experiments (DoEs) and response surface methodology (RSM) offer structured methodologies for studying factor effects and optimizing processing conditions in plastic recycling. For instance, Abdullahi et al. [36] optimized the recycling of LDPE post-consumer household waste by dissolution–precipitation using RSM with a Box–Behnken (BB) design. This study focused on temperature, residence time, and the solvent-to-non-solvent ratio to maximize the recovery yield of PE. Their optimization resulted in a high yield of 93.4% while maintaining minimal degradation of the mechanical properties compared to the virgin material. Similarly, Selvaganapathy et al. [37] employed a BB design to optimize pyrolysis parameters for converting waste polystyrene (PS) into liquid fuel. They identified temperature and residence time as critical parameters and optimized them to enhance liquid fuel yield, achieving notable improvements in both yield and efficiency.

Despite advances in MPPW recycling and the emergence of dissolution–precipitation technology, a gap still exists in the systematic investigation of operating conditions and their influence on the yield and properties of the recovered polymers when dealing with mixed MPPW, particularly realistic streams from recycling centers with added complexity. Furthermore, limited attention has been given to optimizing dissolution–precipitation processes for mixed post-consumer MPPW, considering polymer selectivity and recyclate quality constraints simultaneously.

To address this gap, the present work aims to investigate and optimize the operating conditions of a mixed MPPW dissolution–precipitation process intended for industrial application, using toluene as the solvent. The objective is to recover PE with low polypropylene (PP) contamination (relative composition below 5 wt%). PP negatively affects the mechanical properties of recycled PE [38,39]: (i) ductility is significantly reduced due to the immiscibility and phase separation between the two polymers; and (ii) tensile strength is weakened because of the poor miscibility and consequent inadequate stress distribution, resulting in more brittle materials. By keeping PP content low, interfacial adhesion between PE and PP is maintained, reducing or eliminating the need for compatibilizers, thereby enhancing the potential economic viability of the recycling process.

The entire study is conducted using DoE and RSM tools to investigate the most influential operating conditions for polymer dissolution—namely, temperature, time, solvent-to-waste ratio, and agitation speed—while maintaining the concentration of PP below 5 wt%. Particle size was not included as a factor because the feedstock was already supplied in a relatively small size, and further size reduction had previously been shown to be impractical [40]. These findings provide insights to enhance MPPW management and offer a potentially scalable recycling solution for widespread adoption.

The significance of tackling the recycling of hard-to-recycle plastic waste streams, such as mixed post-consumer MPPW, extends beyond environmental benefits. Economically, it promotes resource efficiency by reducing reliance on virgin materials and decreasing the volume of waste destined for disposal. Strategically, improving the recyclability of MPPW aligns with global sustainability goals and supports the transition towards a circular economy, where materials are continuously reused and recycled.

## 2. Materials and Methods

### 2.1. Materials

Interecycling^®^ (Tondela, Portugal) supplied crushed and washed post-consumer mixed MPPW with an average particle size of approximately 2 cm^2^ and a moisture content of 19 wt%. Before experiments, the MPPW underwent a second washing process with water at 80–90 °C for at least 10 min, and then drying in a ventilated oven at 70 °C until achieving a constant moisture content of 0.61 wt%. Representative samples were collected using the quartering method [41]. Toluene (p.a. grade) from Carlo Erba (Val-de-Reuil, France) was used as a solvent without pretreatment.

### 2.2. Characterization of MPPW

The MPPW was characterized elsewhere [40] by Fourier Transform Infrared Spectroscopy (FTIR), Differential Scanning Calorimetry (DSC), Thermogravimetric Analysis (TGA), and Energy-Dispersive X-ray Spectroscopy (EDS). The analyses revealed a heterogeneous composition. The main families comprised polyolefins, including LDPE, HDPE, and PP; possible polyethylene-based copolymers, such as poly(ethylene-*co*-vinyl acetate) (EVA); PET; polyamide (PA); and polystyrene (PS). Plastic additives, paper, and aluminum were also detected. In terms of abundances, a fractionation using toluene and NMP yielded 50.7 wt% polyolefin-rich fractions (LDPE, HDPE, PP), 20.5 wt% PET-rich fraction, and 8.30 wt% assigned to PS and plastic additives (See Table S1 in the Supplementary Material). Additionally, TGA indicated 6.8 wt% inorganic compounds, and the remaining 13.7 wt% corresponded to unidentified undissolved residues.

### 2.3. Dissolution–Precipitation of MPPW

MPPW underwent dissolution in toluene, selected for its well-established ability to selectively dissolve polyolefins and considering the intended short-term industrial application of the process. In addition, preliminary qualitative laboratory-scale solvent screening experiments were conducted using virgin polymers to support solvent selection. The solvents evaluated included toluene, xylene, tetrahydrofuran, dimethyl sulfoxide, and N-methylpyrrolidone, all p.a. grade from Carlo Erba (Val-de-Reuil, France). A summary of these results is presented in Table S2 in the Supplementary Material.

**Figure 1:**
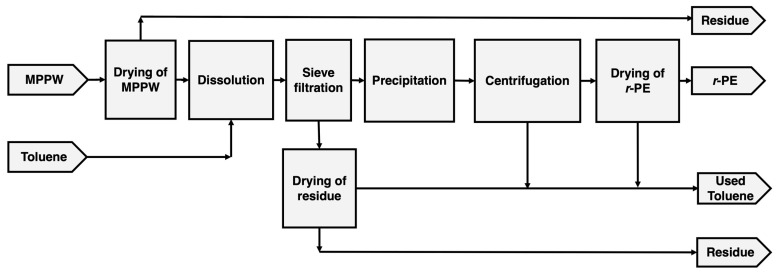
Flowchart of MPPW dissolution–precipitation process carried out in this work.

**Figure 2:**
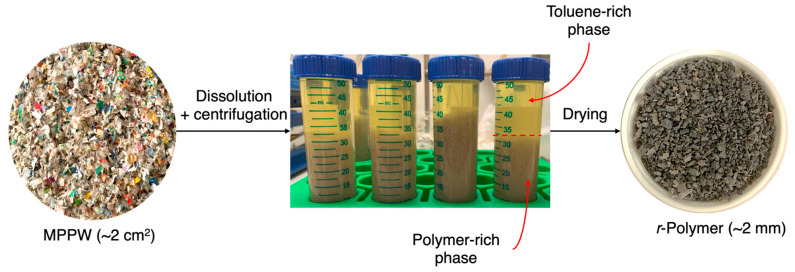
Appearance of the MPPW; solvent-rich and polymer-rich phases after centrifugation, and the recovered *r*-Polymer after drying and milling.

The dissolution–precipitation of MPPW followed the procedure outlined in Figure 1. Considering the health and flammability risks associated with toluene, all experiments involving this solvent were conducted in a chemical fume hood under controlled temperature and agitation, using nitrogen-inertized vessels. MPPW underwent dissolution in toluene in a three-neck round bottom flask (equipped with a condenser, thermometer, nitrogen inlet, and magnetic stirrer) immersed in a silicone bath with agitation and controlled temperature. The dissolution was carried out under N_2_ flow (~42 cm^3^/min) to prevent or minimize thermo-oxidative degradation. Upon dissolution, the extract fraction and the undissolved residue were separated using a stainless-steel coarse sieve with an opening of 500 µm. The dissolved polymer in the extract was then precipitated by cooling it to room temperature, without the addition of any antisolvent. The recovered polymer (*r*-Polymer) was obtained by centrifugation at 10,000 rpm for 15 min to remove most of the solvent and then thoroughly dried in a ventilated oven at 70 °C. The toluene was partially recovered during centrifugation (up to 76 vol% recovery), but it was not reused in this study. The appearance of the recovered polymer and solvent is shown in Figure 2.

### 2.4. Quantitative Analysis via Differential Scanning Calorimetry (DSC)

DSC was employed to quantify LDPE, HDPE, and PP in the *r*-Polymer. The analyses were conducted using a NETZSCH DSC 2024 F1 Phoenix^®^ (NETZSCH-Gerätebau GmbH, Selb, Germany) analyzer using aluminum pans, covering a temperature range from 25 °C to 200 °C at a heating rate of 10 °C/min.

**Figure 3:**
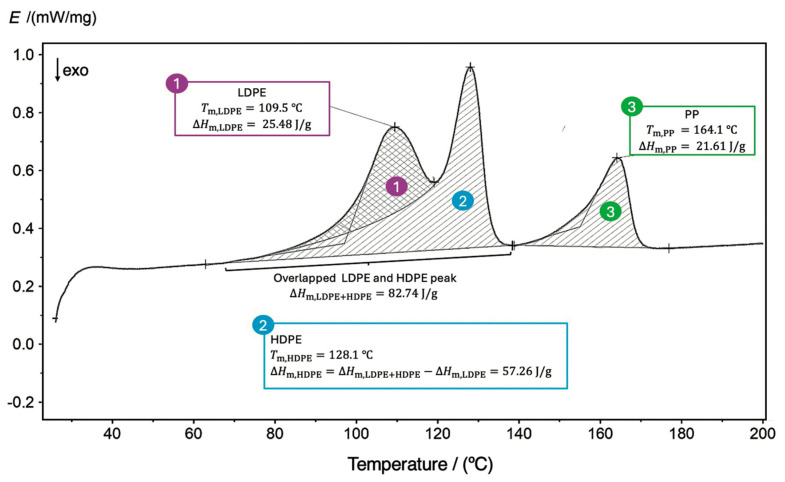
Typical DSC thermogram of samples of *r*-Polymer composed of LDPE, HDPE, and PP collected at 110 °C, 45 min, 700 rpm, and solvent-to-waste ratio of 30 mL/g.

Three typical cases of DSC thermograms were identified during the analysis of *r*-Polymer samples obtained by the process described in Figure 1: the first, where only LDPE is present; the second, where LDPE and HDPE appear as overlapping peaks; and the third, where, in addition to the overlapped peaks of LDPE and HDPE, a third peak of PP is also identified. Figure 3 presents a thermogram for the third and most complex case, which corresponds to the *r*-Polymer obtained at 110 °C, 45 min, 700 rpm, and a solvent-to-waste ratio of 30 mL/g.

The quantitative determination of LDPE, HDPE, and PP comprehended two steps: (i) identifying each polymer peak on the DSC thermogram based on its characteristic melting temperature ($T_{m}$); and (ii) calculating the melting enthalpy per gram of analyzed sample that is consumed by each polymer ($\Delta H_{m,i}$) in the mixture by the following:

(1) $$\Delta H_{m,\;i}=\frac{1}{a}\int_{T_{1}}^{T_{2}}E_{i}\cdot dT$$

where a is the heating rate (10 °C/min) and $E_{i}$ represents the energy absorbed during the melting of polymer i between temperatures $T_{1}\;$ and $T_{2}$, per gram of the analyzed sample.

The conventional method for determining the melting enthalpy involves performing a first DSC run to erase the thermal history of the sample, followed by a second run from which the melting enthalpy is determined. However, in this work, the first run was intentionally used to account for the changes in crystallinity induced by the dissolution–precipitation process. To quantify the amounts of LDPE and HDPE, the partial area corresponding to the melting of LDPE was determined using the partial area function of the NETZSCH Proteus^®^ 8.0 software. Worth noting is that a comparison between peak deconvolution and partial-area integration was performed using polymeric synthetic mixtures with known compositions. The partial-area calculation yielded results that were closer to the real composition and was therefore adopted for quantification. To account for the low-temperature tail of the HDPE peak, as addressed by other authors [42,43], the Bezier baseline function available in the NETZSCH Proteus^®^ 8.0 software was used. The calculation of the area is illustrated in Figure 3, with the LDPE area labeled as number 1. The HDPE area (number 2 in Figure 3) was then determined by subtracting the LDPE partial area from the total combined area.

The mass fraction of each polymer $\left(w_{i}^{\prime }\right)$ in the *r*-Polymer was subsequently computed by the following:

(2) $$w_{i}^{\prime }=100\times \frac{\Delta H_{m,\;i}}{\Delta H_{m,\;i}^{\mathrm{ref}}}$$

where $\Delta H_{m,i}$ is the melting enthalpy of each polymer on the *r*-Polymer, and $\Delta H_{m,\;i}^{\mathrm{ref}}$ is the reference melting enthalpy of polymer i obtained from the corresponding fractions isolated from MPPW as explained next.

The reference values of the melting enthalpy of each polymer, $\Delta H_{m,\;i}^{\mathrm{ref}}$, were obtained from LDPE, HDPE, and PP-rich fractions isolated from MPPW through a multi-step dissolution process in toluene, as detailed in Table S1 in the Supplementary Material. The LDPE-rich fraction, isolated at 72 °C, exhibited a melting enthalpy of 107.1 J/g. Additionally, the LDPE/HDPE blend was isolated at 100 °C, and it was determined that it contained 33.6 wt% of LDPE and 66.4 wt% of HDPE, with a melting enthalpy of 133.9 J/g for the HDPE. Similarly, it was determined that the HDPE/PP blend, isolated at 110 °C, contained 61.0 wt% of HDPE and 39.0 wt% of PP, with a melting enthalpy of 101.4 J/g for the PP. These values of 107.1 J/g, 133.9 J/g, and 101.4 J/g were used as the reference melting enthalpies for LDPE, HDPE, and PP, respectively, present in the MPPW.

The mass fractions $w_{i}^{\prime }$ were then normalized to express the *r*-Polymer composition in terms of LDPE, HDPE, and PP only, i.e., disregarding non-identified plastics additives and possible PS appearing in the *r*-Polymer after the dissolution–precipitation process. Accordingly, the relative abundances ($w_{i})$ of each polymer were obtained by the following:

(3) $$w_{i}=100\times \frac{w_{i}^{\prime }}{w_{\mathrm{LDPE}}^{\prime }+w_{\mathrm{HDPE}}^{\prime }+w_{\mathrm{PP}}^{\prime }}\;\mathrm{and}\;\sum_{i=1}^{3}w_{i}=100$$

To illustrate the quantification method, an example based on the experiment conducted at 110 °C, 45 min, 700 rpm, and 30 mL/g (thermogram of Figure 3) is presented. Two overlapping peaks are observed at 109.5 °C and 128.1 °C, corresponding to LDPE and HDPE, respectively, and the PP peak occurs at 164.1 °C. The combined enthalpy of LDPE and HDPE was calculated from the area under the overlapping peaks using Equation (1), giving rise to $\Delta H_{m,\mathrm{LDPE}+\mathrm{HDPE}}=$ 82.74 J/g. The enthalpy of LDPE was obtained using the partial area function mentioned above, yielding $\Delta H_{m,\mathrm{LDPE}}=\;25.48$ J/g. The melting enthalpy of HDPE is calculated by difference: $\Delta H_{m,\mathrm{HDPE}}=\;82.74-25.48=57.26$ J/g. Finally, the melting enthalpy of PP obtained by integration of the third peak is $\Delta H_{m,\mathrm{PP}}=$ 21.61 J/g. The mass fraction of each polymer calculated from Equation (2) is $w_{\mathrm{LDPE}}^{\prime }=23.8$ wt%, $w_{\mathrm{HDPE}}^{\prime }=42.8$ wt% and $w_{\mathrm{PP}}^{\prime }=21.3$ wt% (the remaining 12.1 wt% are unknown components, possibly plastic additives). The normalized concentrations of LDPE, HDPE, and PP calculated with Equation (3) are 27.1 wt%, 48.7 wt%, and 24.3 wt%.

### 2.5. Additional Characterization of r-Polymer

FTIR spectra were obtained using a Perkin Elmer (Waltham, MA, USA) system with a universal ATR accessory, averaging 64 scans at 4.0 cm^−1^ resolution over the 400–4000 cm^−1^ range, under controlled conditions (23 °C and 35 % relative humidity). 

X-ray diffractograms were recorded using an Empyrean (Malvern Panalytical, Almelo, Netherlands) diffractometer with Cu Kα radiation (λ ≈ 1.54060 Å), operating at 45 kV and 40 mA. Data were collected using a step size of 0.026°/s over a 3°–50° range.

Solid-state ^13^C cross-polarization magic angle spinning nuclear magnetic resonance (CP-MAS NMR) spectra were acquired using a Bruker Avance III 400 MHz (Bruker BioSpin GmbH, Rheinstetten, Germany) spectrometer, with 7000 scans, a 90° proton pulse, a 1 ms contact time, and a 2.5 s delay.

### 2.6. Design of Experiments (DoEs) and Response Surface Methodology (RSM)

The optimization of the dissolution–precipitation process used a combination of DoE and RSM. RSM comprises a set of statistical and mathematical techniques employed to develop, improve, and optimize processes by systematically evaluating the effects of multiple independent variables and their interactions on a response of interest. The Box–Behnken (BB) design was selected based on its efficiency and practical applicability with a small number of experiments over other designs like the full factorial or central composite designs [36,37].

The design used in this study assessed the influence of four factors (dissolution temperature, dissolution time, solvent-to-waste ratio, and agitation speed) on the total dissolution yield, and the PE and PP composition of the *r*-Polymer. Each factor was tested at three levels, including a central point. Each experiment was replicated to address sample heterogeneity and ensure the reliability of the results, resulting in 25 independent pairs of experiments (50 experiments in total) conducted in a randomized sequence to minimize systematic errors. Table 1 presents the levels for each factor tested in this study. The complete list of fifty experiments, along with their respective level combinations, is detailed in Table S3 of the Supplementary Material.

The total dissolution yield ($\eta_{\mathrm{Total}}$) was determined using Equation (4), while the concentrations of PE ($w_{\mathrm{PE}}$) and PP ($w_{\mathrm{PP}}$) were calculated using Equations (2) and (3), with the PE content combining the contributions of LDPE and HDPE.

(4) $$\eta_{\mathrm{Total}}=100\times \frac{m_{r-\mathrm{Polymer}}}{m_{\mathrm{MPPW}}}$$

Here $m_{r-\mathrm{Polymer}}$ is the total mass of the *r*-Polymer, and $m_{\mathrm{MPPW}}$ is the mass of the initial MPPW.

The data fitting employed a second-order polynomial model to elucidate linear, quadratic, and interaction effects:

(5) $$Y=b_{0}+\;\overset{4}{\underset{j=1}{\sum }}b_{j}X_{j}+\overset{4}{\underset{j=1}{\sum }}b_{\mathrm{jj}}X_{j}^{2}\;+\;\overset{4}{\underset{j=1}{\sum }}\overset{4}{\underset{k>1}{\sum }}b_{\mathrm{jk}}X_{j}X_{k}$$

(6) $$X_{j}=\frac{x_{j}-x_{j,0}}{\Delta x_{j}}$$

where $b_{0}$ is a constant, $b_{j}$, $b_{\mathrm{jj}}$, and $b_{\mathrm{jk}}$ are the model coefficients for the linear, quadratic, and interaction effects, respectively, $X_{j}$ and $x_{j}$ represent the coded and the real values of the independent variable or factor j, $x_{j,0}$ is the value of the central point of the independent variable j, and $\Delta x_{j}$ is the step change.

The statistical analysis was conducted using JMP^®^ 18.0 software. Analysis of Variance (ANOVA) was used to assess the significance of the effects via Fisher’s test, with p-values indicating the probability of the Fisher statistic, $\left(p\left(F\right)\right)$. The significance of the model coefficients was evaluated using *t*-tests.

The goodness of fit for the regression models was quantified by the coefficients of determination, $\left(R^{2}\right)$, adjusted coefficients of determination, $\left(R_{\mathrm{adj}}^{2}\right)$, and the average absolute relative deviation (AARD) is defined by the following:

(7) $$\mathrm{AARD}=\frac{1}{n}\;\overset{n}{\underset{i=1}{\sum }}\left|\frac{\;y_{i}^{\exp}-y_{i}^{\mathrm{pred}}}{y_{i}^{\exp}}\right|$$

where n is the number of experiments, $y_{i}^{\exp}$ is the experimental value, and $y_{i}^{\mathrm{pred}}$ is the predicted value.

## 3. Results and Discussion

To optimize the dissolution process, the BB design combined with RSM was adopted to investigate the influence of dissolution temperature, dissolution time, solvent-to-waste ratio, and agitation speed on the following key performance indicators: total dissolution yield ($\eta_{\mathrm{Total}}$), relative PE composition ($w_{\mathrm{PE}})$, and relative PP composition ($w_{\mathrm{PP}}$).

### 3.1. Total Dissolution Yield

**Figure 4:**
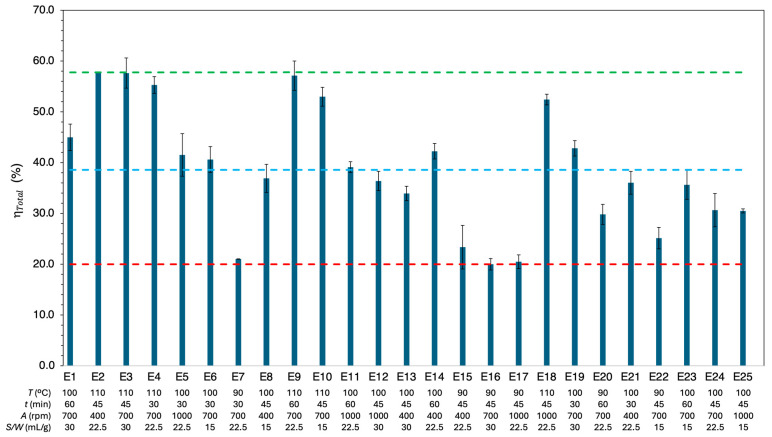
Total dissolution yield ($\eta_{\mathrm{Total}}$) of all experiments against experimental conditions. Blue dashed line indicates the global average yield (38.6%), green dashed line is the maximum yield (57.8%), and red dashed line is the minimum yield (20.0%). Error bars denote the standard deviation.

Figure 4 depicts the total dissolution yield, $\eta_{\mathrm{Total}}$, for each experimental condition. Detailed results for all fifty experiments are provided in Table S3 in the Supplementary Material. The results ranged from a minimum of 20.0% (90 °C, 45 min, 700 rpm, 30 mL/g) to a maximum of 57.8% (110 °C, 45 min, 700 rpm, 30 mL/g), with an average of 38.6%. The maximum relative deviation between replicates was 18.4%, further highlighting the heterogeneous nature of the MPPW. Additionally, the maximum yield was comparable to the total extractable material from MPPW using toluene, which had previously been determined to be 59.0% (see Table S1 in the Supplementary Material).

A comparative analysis highlighted the significant influence of various factors on $\eta_{\mathrm{Total}}$. However, the dissolution temperature proved to be crucial, with higher temperatures (110 °C) consistently resulting in higher yields between 55.1% and 57.8%, compared to those conducted at the lowest temperature (90 °C), which yields ranged from 20.0% to 29.8%.

Temperature affects both the thermodynamic and kinetic properties of polymer dissolution. Thermodynamically, the process is governed by the Gibbs free energy of mixing ($\Delta G_{\mathrm{mix}}$), which results from the interplay between the enthalpy change ($\Delta H_{\mathrm{mix}}$) and the entropy change ($\Delta S_{\mathrm{mix}}$) of mixing [44]. Dissolution occurs spontaneously when $\Delta G_{\mathrm{mix}}$ is negative; at $\Delta G_{\mathrm{mix}}$ = 0, the system is at equilibrium. As temperature increases, the entropic contribution $\left(-T\Delta S_{\mathrm{mix}}\right)$ becomes more significant, and, when $\Delta S_{\mathrm{mix}}$ is positive, this can make dissolution more thermodynamically favorable. Moreover, the Flory–Huggins theory further explains the miscibility between a polymer and a solvent, and the Flory–Huggins interaction parameter, $\chi_{12}$, measures the interaction between the solvent (1) and the polymer (2) molecules. This parameter decreases as temperature increases, indicating improved solubility [44].

From a kinetic perspective, higher temperatures improve the mutual diffusion between the polymer and the solvent, leading to more efficient mass transfer and consequent higher dissolution rates [44]. This behavior can be associated with increased chain mobility and the presence of additional free volume at elevated temperatures, which facilitates solvent penetration into the polymer matrix and accelerates the dissolution process [44].

The influence of dissolution time on $\eta_{\mathrm{Total}}$ was mainly observed at lower temperatures, as demonstrated by the comparison of samples E7 and E20. At these temperatures, the dissolution rate decreased due to reduced polymer chain mobility in the gel phase and solvent diffusivity in the solid phase. Under these conditions, a longer induction time was required, i.e., the time needed for the polymer to initiate dissolution [45], resulting in a longer dissolution time to approach equilibrium. In contrast, at higher temperatures, polymer chains exhibit greater segmental mobility, and solvent diffusivity is enhanced. As a result, the system approaches equilibrium more rapidly, reducing the influence of prolonged dissolution time on the overall dissolution yield.

Changes in agitation speed led to minor variations in $\eta_{\mathrm{Total}}$ when the speed was increased from 400 rpm to 1000 rpm, as observed when comparing samples E5 and E21, E11 and E14, as well as E15 and E17. This occurred because agitation initially enhanced mass transfer by reducing the thickness of the gel layer formed on the polymer surface. However, this effect reaches a limit where further increases in agitation no longer significantly improve the dissolution rate [27]. The minimal changes in $\eta_{\mathrm{Total}}$ observed between 400 rpm and 1000 rpm across various temperatures, dissolution times and solvent-to-waste ratios suggested that 400 rpm was sufficient for effective dissolution, with higher agitation speeds offering no substantial additional benefit.

Similarly, samples E3 and E10, as well as E6 and E19, exhibited slight increases in $\eta_{\mathrm{Total}}$ when the solvent-to-waste ratio was increased from 15 mL/g to 30 mL/g. Increasing the solvent-to-waste ratio results in more dilute bulk solutions, which enhances the driving force for mass transfer by maintaining a higher concentration gradient between the polymer surface and the bulk solution. Moreover, the decreased viscosity of the polymer solution increases the convective mass transfer coefficient, which further improves the dissolution rate. Consequently, a higher solvent-to-waste ratio reduced the formation of the gel layer at the polymer-solvent interface and enhanced mass transport. These observations corroborated previous studies on polymer dissolution in organic solvents, which showed that higher solvent concentrations improved solvent diffusion into the polymer matrix and led to faster dissolution rates. However, further increases in the solvent-to-waste ratio yielded only marginal improvements [45,46].

**Figure 5:**
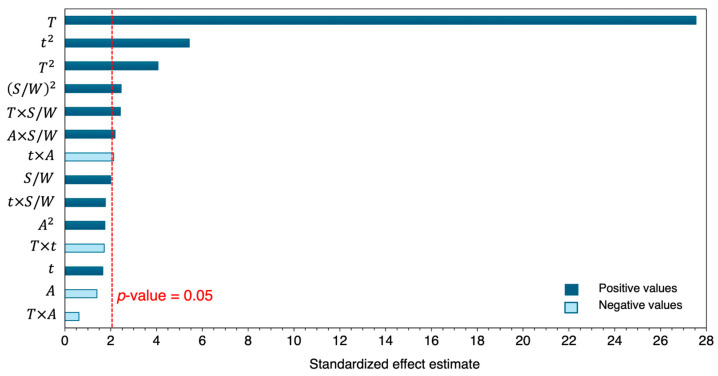
Pareto chart of the total dissolution yield ($\eta_{\mathrm{Total}}$) response according to different operating variables. Dark bars denote positive contribution (desired effect), and light bars denote negative contribution (undesired effect). The red vertical line corresponds to the significance level at a 95% confidence interval.

Following the application of RSM, the data collected were modeled to identify factors influencing the $\eta_{\mathrm{Total}}$. ANOVA was used to evaluate the significance of the effects, with a p-value ≤ 0.05 serving as the criterion for significance at 95% confidence. The Pareto Chart of $\eta_{\mathrm{Total}}$, depicted in Figure 5, highlights temperature as a critical variable; its linear $\left(T\right)$ and quadratic $\left(T^{2}\right)$ effects were found to be significant, emphasizing its essential role in the dissolution process. Similar results were reported in the optimization of waste LDPE dissolution by Abdullahi et al. [36], where temperature, including its quadratic effect, was the most significant factor. Additionally, our results align with studies of other polymer/solvent systems [47,48].

As regards the dissolution time, the analysis highlighted the quadratic effect of time $\left(t^{2}\right)$ on dissolution, suggesting that there was an optimal time point that maximized yield. Exceeding this time could lead to disadvantages, such as polymer degradation or solvent loss, reinforcing the need for careful optimization of the process. Similarly, the quadratic effect of the solvent-to-waste ratio, ${\left(S/W\right)}^{2}$, was identified as significant, confirming the influence of solvent-to-waste ratio on the dissolution rate, as previously observed in the dissolution of thermoplastic polymers by Achilias et al. [48] and Mumbach et al. [49].

Interaction effects like those between temperature and solvent-to-waste ratio $\left(T\times \left(S/W\right)\right)$, agitation speed and solvent-to-waste ratio $\left(A\times \left(S/W\right)\right)$, and time and agitation speed $\left(t\times A\right)$ were found to significantly influence $\eta_{\mathrm{Total}}$, with all except the interaction between time and agitation speed showing positive impacts. The fact that the interactions between temperature and solvent-to-waste ratio, as well as agitation speed and solvent-to-waste ratio, were significant indicated synergetic effects from enhanced solvent diffusion, polymer chain mobility and convective mass transfer, which together improve the dissolution process. On the contrary, the negative effect of time and agitation interaction suggested that prolonged agitation, in an inadequately sealed system, such as the laboratory setup used in this work, could lead to solvent evaporation, negatively impacting the dissolution process.

A comprehensive assessment of the model’s performance metrics confirmed the reliability of the results. Table 2 presents the regression coefficients of the full model, detailing the p-values and the resulting statistical parameters. The *p*-values from the *t*-test for each effect coefficient confirmed the statistical significance (or lack thereof) observed in the Pareto chart. The full model achieved a strong fit, evidenced by statistics $R^{2}=\;0.959$, $R_{\mathrm{adj}}^{2}=\;0.943$*,* and AARD= 5.52%. This indicates that 95.9% of data variability is explained by the model. Subsequently, the reduced model was developed, retaining only the significant factors and interactions. The reduced model continues to demonstrate high accuracy with $R^{2}=0.938$, $R_{\mathrm{adj}}^{2}=0.928$, and AARD=6.94%, confirming a strong correlation and a good fit with fewer factors. The obtained reduced model is presented in Equation (8). For more details, Table S4 in the Supplementary Material compares the coded coefficients and fitting assessment parameters of the full and reduced models.

(8) $$\eta_{\mathrm{Total}}=361-6.20\;T+0.0354\;T^{2}-1.74\;t+0.0231\;t^{2}-5.24\;\left(S/W\right)+0.0284\;{\left(S/W\right)}^{2}-0.000638\;A\;+0.0327\;T\;\left(S/W\right)-0.000479\;t\;A+0.000986\;\left(S/W\right)\;A\;\pm \;3.01$$

**Figure 6:**
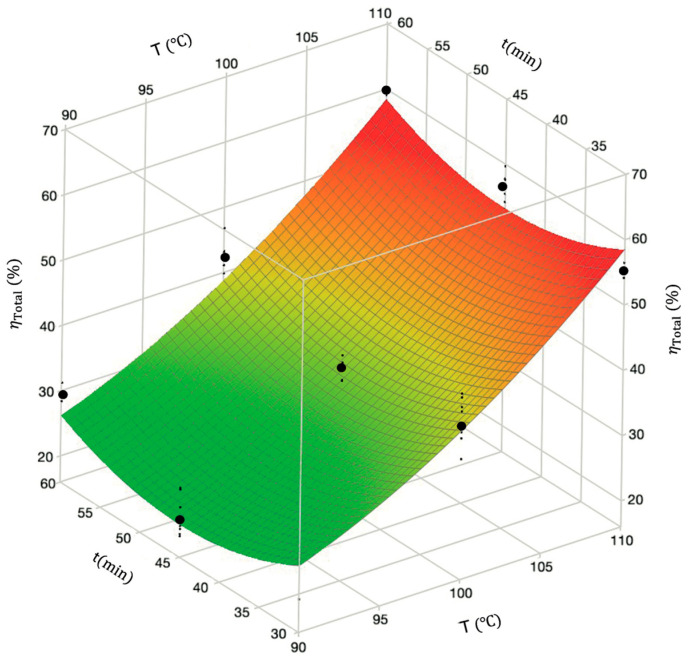
Response surface of total dissolution yield as a function of temperature and time at fixed agitation speed (700 rpm) and solvent-to-waste ratio (22.5 mL/g). Black dots are experimental data, and $\eta_{\mathrm{Total}}\;\left(T,\;t\right)$ surface is given by Equation (8) (reduced model).

The surface profile of the total dissolution yield given by the reduced model as a function of temperature (the most significant factor) and time ($\eta_{\mathrm{Total}},\;\left(T,\;t\right)$) is illustrated in Figure 6 at fixed agitation speed (700 rpm) and solvent-to-waste ratio (22.5 mL/g), while $\eta_{\mathrm{Total}}\;\left(T,\;A\right)$ and $\eta_{\mathrm{Total}}\;\left(T,(S/W\right))$ are shown in Figure S1 in the Supplementary Material. These plots highlighted the prominent influence of temperature on the yield. The optimum conditions for $\eta_{\mathrm{Total}}$ were 110 °C, 30 min, 1000 rpm, and 30 mL/g.

### 3.2. Relative Content of PE in the Recovered Polymer, $w_{PE}$

The concentration of PE and PP in the *r*-Polymer was determined by thermal analysis using DSC and the calculation procedure described in Section 2.4.

**Figure 7:**
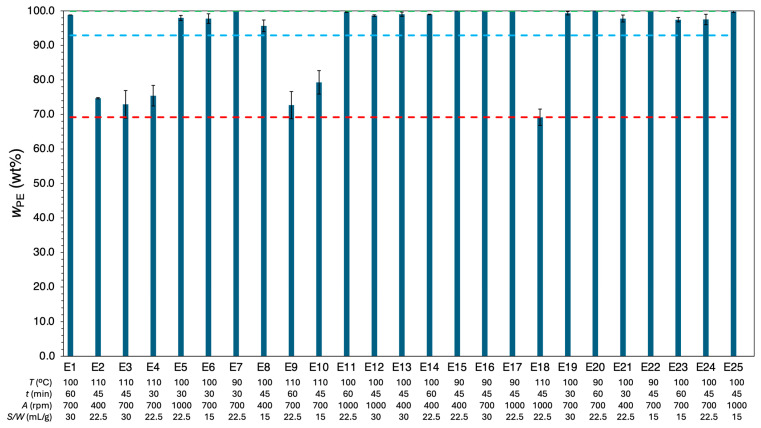
Relative content of PE ($w_{\mathrm{PE}},\;\mathrm{wt}\%)\;$ for experiments E1 to E25 as a function of the experimental conditions. Light blue dashed line indicates the global average of $w_{\mathrm{PE}}$ (92.9 wt%), green dashed line is the maximum $w_{\mathrm{PE}}$ (100 wt%), and the red dashed line is the minimum $w_{\mathrm{PE}}$ (69.2 wt%). Error bars correspond to the standard deviation.

Figure 7 shows the relative content of PE in the *r*-Polymer $(w_{\mathrm{PE}}$, wt%) for each set of twenty-five experimental conditions, along with their corresponding standard deviations. Detailed $w_{\mathrm{PE}}$ values for all fifty experiments are provided in Table S3 in the Supplementary Material. The $w_{\mathrm{PE}}$ results in Figure 7 ranged from a minimum of 69.2 wt% (110 °C, 45 min, 1000 rpm, 22.5 mL/g) to a maximum of 100 wt% (achieved under several operating conditions), with a global average of 92.9 wt% and maximum relative deviation between replicates of 5.5%.

Temperature was the most important factor influencing the composition of the recovered material, with PE being the most prominent component of the *r*-Polymer. Notably, higher temperatures consistently yielded lower $w_{\mathrm{PE}}$. At 90 °C, the dissolution process predominantly yielded LDPE, resulting in a consistent $w_{\mathrm{PE}}$ of 100 wt%, as observed in experiments E7, E15, E16, E17, and E20, regardless of dissolution time, agitation speed, or solvent-to-waste ratio. As the temperature increased to 100 °C, LDPE, HDPE, and PP dissolved with $w_{\mathrm{PE}}$ ranging from 95.7 wt% to 99.8 wt%. At 110 °C, $w_{\mathrm{PE}}$ decreased ranging from 69.2 wt% to 79.3 wt%, with more PP being dissolved.

Although toluene is a good solvent for both LDPE and HDPE, their dissolution behaviors differ due to variations in crystallinity. LDPE’s branched structure and higher amorphous content provide increased chain mobility and free volume, and weaker intermolecular forces, making solvent diffusion and chain disentanglement easier. This allows LDPE to dissolve at relatively lower temperatures [50]. In contrast, HDPE has a more linear structure, resulting in tightly packed polymer chains and higher crystallinity [51]. This high crystallinity requires more energy to break the intermolecular forces within the crystalline domains, and thus a higher dissolution temperature. Additionally, HDPE typically has a much higher molar mass (typically $\sim 10^{6}$ g/mol, compared to LDPE’s up to $4\times 10^{5}$ g/mol [52]), which further slows the dissolution rate. The higher molar mass increases chain entanglement, making it more difficult and time-consuming for the polymer to fully disentangle and dissolve, as discussed by Valois et al. [53].

Dissolution time appeared to exert a slight negative impact on $w_{\mathrm{PE}}$, as evidenced by a decrease of up to 3.6% when comparing samples E1 and E19, as well as E4 and E9. However, due to the overlapping error bars, this variation cannot be conclusively attributed to dissolution time alone.

The solvent-to-waste ratio had minimal influence on $w_{\mathrm{PE}}$, though its small effect can be either negative or positive depending on the temperature. At 110 °C, increasing the solvent-to-waste ratio from 15 mL/g to 30 mL/g resulted in 8.0% decrease in $w_{\mathrm{PE}}$, as seen in experiments E10 and E3. On the other hand, at the moderate temperature of 100 °C, the same increase in the solvent-to-waste ratio led to a 1.6% rise in $w_{\mathrm{PE}}$, as demonstrated by experiments E6 and E19. This dual effect of the solvent-to-waste ratio is due to reduced selectivity of PE over PP at higher dissolution temperatures [30,33] and increased the overall dissolution rate. Thus, the interaction between the solvent-to-waste ratio and temperature suggests that optimizing $w_{\mathrm{PE}}$ requires balancing these factors.

Similarly, the influence of agitation speed on the *r*-Polymer composition was not straightforward, as it showed opposite tendencies depending on the temperature and the solvent-to-waste ratio. At lower temperatures or at a lower solvent-to-waste ratio, increasing agitation speed generally led to higher $w_{\mathrm{PE}}$. In contrast, at higher temperatures or at a higher solvent-to-waste ratio, higher agitation speed tended to decrease $w_{\mathrm{PE}}$. For instance, at 110 °C, increasing the agitation from 700 rpm to 1000 rpm reduced $w_{\mathrm{PE}}$ from a range of 69.9–77.5 wt% to 67.5–70.9 wt%. This behavior is attributed to stronger mass-transfer effects induced by agitation, which decreases the selectivity toward PE.

Overall, $w_{\mathrm{PE}}$ was found to be inversely proportional to $\eta_{\mathrm{Total}}$. This indicates that as $h_{\mathrm{Total}}$ increases, the proportion of PE on the recovered material decreases. This relationship implies that conditions promoting higher dissolution yields may preferentially dissolve other components than PE, decreasing its relative content in the final *r*-Polymer mixture. Furthermore, under more severe conditions, most PE may already have been dissolved, allowing the dissolution process to extend to other components present in the MPPW.

Likewise, for the $\eta_{\mathrm{Total}}$ model, ANOVA results for the $w_{\mathrm{PE}}$ response confirmed the significance of temperature, both in its linear (T) and quadratic $\left(T^{2}\right)$ terms, as well as its interaction with the solvent-to-waste ratio $\left(T\times \left(S/W\right)\right)$, with the linear effect of temperature being the most significant. As with $\eta_{\mathrm{Total}}$, all significant effects negatively influenced $w_{\mathrm{PE}}$, indicating that higher dissolution yields correlated with lower $w_{\mathrm{PE}}$ values. These findings reinforce the critical role of temperature in the process and confirm that lower temperatures are more favorable for maximizing $w_{\mathrm{PE}}$. Further details, including the Pareto chart of $w_{\mathrm{PE}}$ response can be consulted in Figure S2 in the Supplementary Material.

Table 3 compares the full and the reduced models of $w_{\mathrm{PE}}$ response, including the number of terms of the model, $R^{2}$, $R_{\mathrm{adj}}^{2}$, and AARD. The full model achieved a robust fit and prediction accuracy, reporting $R^{2}=$ 0.974, $R_{\mathrm{adj}}^{2}=\;0.964$, and AARD = 1.69%. The *t*-tests confirmed the statistical significance of the effects identified in the Pareto chart analysis (Figure S2 in Supplementary Material). Notably, the full model accounts for 97.4% of the variability in $w_{\mathrm{PE}}$ reflecting a high degree of accuracy with only a 1.69% average deviation from experimental data. The reduced and uncoded $w_{\mathrm{PE}}$ model, given by Equation (9), reached $R^{2}=\;0.967$, $R_{\mathrm{adj}}^{2}=$ 0.965 and AARD = 1.62%. These statistics indicate that the reduced model, with only four terms, achieves an accuracy similar to the full model, which contains 15 terms. For more details, Table S5 in the Supplementary Material presents the coded coefficients and fitting assessment parameters for the full and reduced models.

(9) $$w_{\mathrm{PE}}\left(\mathrm{wt}\%\right)=-952+21.8\;T-0.113\;T^{2}-0.0213\;T\;\left(S/W\right)+2.13\;\left(S/W\right)\;\pm \;1.09$$

**Figure 8:**
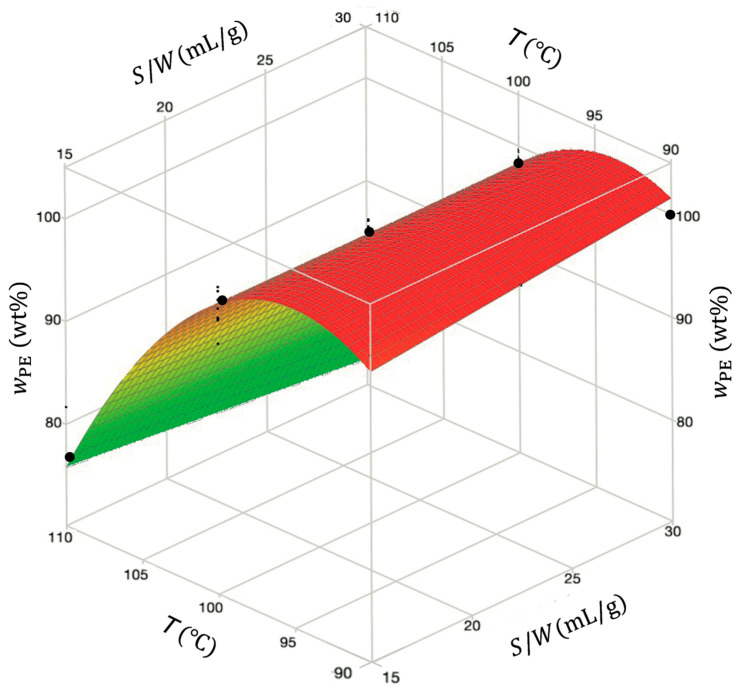
Response surface obtained for PE content as a function of temperature and solvent-to-waste ratio at fixed time (45 min) and agitation (700 rpm). Black dots are experimental data, and $w_{\mathrm{PE}}\;\left(T,\;\left(S/W\right)\right)$ surface is given by Equation (9) (reduced model).

Figure 8 illustrates the response surface given by the reduced model of $w_{\mathrm{PE}}$ as a function of temperature and solvent-to-waste ratio. The $w_{\mathrm{PE}}$ curvature in the plot was attributed to the temperature’s quadratic effect, demonstrating the strong dependence on this variable. Conversely, the solvent-to-waste ratio $\left(S/W\right)$ had a much smaller effect on $w_{\mathrm{PE}}$. The optimum conditions maximizing $w_{\mathrm{PE}}$ were identified at a temperature of 90 °C and a solvent-to-waste ratio of 30 mL/g, as determined by the optimization of the model considering the minimal agitation speed and time of 400 rpm and 30 min, respectively.

### 3.3. Relative Content of PP in the Recovered Polymer, $w_{PP}$

**Figure 9:**
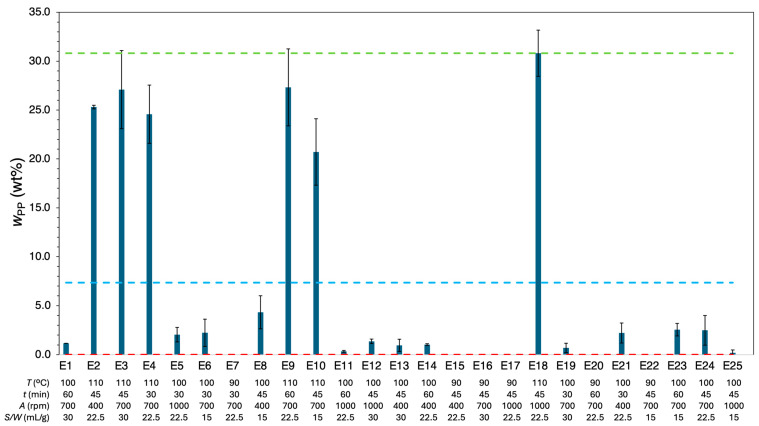
Relative content of PP $\left(w_{\mathrm{PP}},\mathrm{wt}\%\right)\;$ for experiments E1 to E25 as a function of the experimental conditions. Light blue dashed line indicates the global average of $w_{\mathrm{PP}}$ (7.30 wt%), green dashed line is the maximum $w_{\mathrm{PP}}$ (30.8 wt%), and red dashed line is the minimum $w_{\mathrm{PP}}$ (0.0 wt%). Error bars correspond to the standard deviation.

Figure 9 illustrates the relative PP content in the *r*-Polymer $(w_{\mathrm{PP}}$, wt%) for each set of twenty-five experimental conditions, along with their standard deviations. Table S1 in the Supplementary Material provides a comprehensive list of the fifty experiments. At the highest temperature (110 °C), $w_{\mathrm{PP}}$ varied between 20.7 wt% and 30.8 wt%. At 100 °C, the obtained values were between 0.20 wt% and 4.3 wt%.

Conversely, at the lowest temperature (90 °C), the presence of PP on the recovered material was not detected. The global average $w_{\mathrm{PP}}$ was 7.10%, with a maximum relative deviation between replicates of 141%. High relative deviations between replicates were found mostly in small $w_{\mathrm{PP}}$ values observed in experiments conducted at lower temperatures (e.g., experiment E25 for which $w_{\mathrm{PP}}=$ 0.20 ± 0.29 wt%).

The data exhibited a strong correlation between temperature and PP content. While the HDPE dissolution is strongly influenced by its high crystallinity, dense chain packing and high molar mass, PP dissolution is challenged by its crystallinity and stereochemistry. PP has a semi-crystalline structure with methyl side groups that create steric hindrance and increase its rigidity. Due to these factors, PP requires high temperatures to dissolve in toluene. However, once its steric hindrance and crystalline domains are overcome, PP dissolves faster than HDPE because of its lower chain entanglement and typical lower molar mass (up to 6 × 10^5^ g/mol [52]). Thus, PP dissolves more efficiently than HDPE in toluene once the temperature is high enough to break down its crystallinity and steric barriers. The differences observed in the dissolution behavior between PP and the previously discussed LDPE and HDPE agree with reported results of Achilias et al. [33] and Hadi et al. [54], who investigated the dissolution of these polymers in various solvents, including toluene.

At lower temperatures, such as 90 °C, the energy provided was insufficient to break the intermolecular forces and disrupt the crystalline structure, which explains the fact that at 90 °C, no PP was detected in the recovered material. In contrast, at higher temperatures, such as 110 °C, the increased energy provided allows the disruption of crystalline regions and overcomes steric barriers, promoting PP dissolution and consistently exceeding the 5 wt% threshold. At 100 °C, the PP content remains below 5 wt%, which is desirable for the intended MPPW recycling process as it enables efficient PE recovery while keeping PP levels within acceptable limits. A threshold of 5 wt% PP is considered the maximum tolerable level in recycled PE to maintain mechanical performance, as reported by Karaagac et al. [38,39].

Regarding other factors such as agitation, time, and solvent-to-waste ratio, they were not statistically different within the range of values studied. The observations for the PP content in the *r*-Polymer closely complemented those for PE, which was expected, given the assumption $w_{\mathrm{PP}}+w_{\mathrm{PE}}=100$.

The Pareto chart summarizes the influence of the different effects on the $w_{\mathrm{PP}}$ response can be consulted in Figure S3 in the Supplementary Material. The statistical analysis identified both T and $T^{2}$ as significant effects, with the linear effect being the most prominent. Moreover, the interaction between temperature and solvent-to-waste ratio $\left(\;T\times \left(\frac{S}{W}\right)\right)$ also significantly enhanced $w_{\mathrm{PP}}$. These findings confirmed the previously mentioned experimental observations.

Table 4 compares the full and reduced models of $w_{\mathrm{PP}}$ response, including the number of terms of the model, $R^{2}$, $R_{\mathrm{adj}}^{2}$, and AARD. The full model offered a good fit, with $R^{2}=$ 0.974 and $R_{\mathrm{adj}}^{2}=\;0.964$, though high deviations were obtained with AARD = 44.7%. Such a high AARD was due to the contribution of the individual deviations conveyed by the very small $w_{\mathrm{PP}}$ values, which was expected in advance.

Despite the poor predictive capacity of the full model in the range of small $w_{\mathrm{PP}}$ values, a reduced model, including only the significant effects $\left(T,\;T^{2},T\times \left(S/W\right)\right)$, was obtained and demonstrated equivalent behavior, with $R^{2}=0.967$, $R_{\mathrm{adj}}^{2}\;=0.965\;$ and AARD =46.1%. Hence, it was selected for its simplicity. The final reduced and uncoded $w_{\mathrm{PP}}$ model is given by Equation (10), and its response surface as a function of temperature and solvent-to-waste ratio is depicted in Figure 9. In Table S6 in the Supplementary Material, the codified coefficients and fitting parameters of both full and reduced models are listed.

(10) $$w_{\mathrm{PP}}\left(\mathrm{wt}\%\right)=1052-21.8\;T+0.113\;T^{2}+0.0213\;\left(S/W\right)\;T-2.13\;\left(S/W\right)\;\pm \;1.09$$

**Figure 10:**
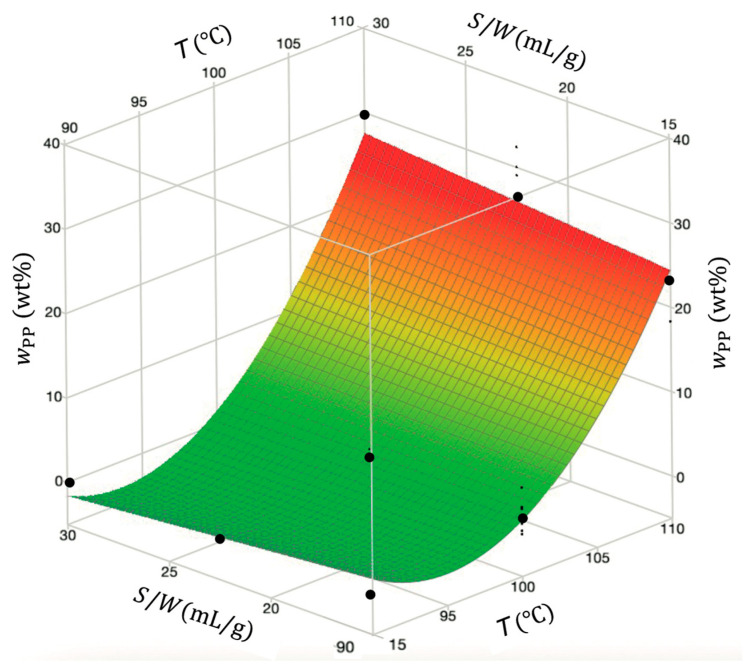
Response surface obtained for PP content as a function of temperature and solvent-to-waste ratio at fixed time (45 min) and agitation speed (700 rpm). Black dots are experimental data, and $w_{\mathrm{PP}}\;\left(T,\;\left(S/W\right)\right)$ surface is given by Equation (10) (reduced model).

The response surface (Figure 10) clearly shows that temperature significantly influences $w_{\mathrm{PP}}$ in the recovered material, while the solvent-to-waste ratio has a minor influence. Higher temperatures contribute to increased PP contamination.

The dominant effect of temperature arises from its strong influence on polymer solubility and on the rate-limiting steps of PP dissolution, such as solvent diffusion into the polymer matrix, disruption of crystalline domains, polymer chain disentanglement, and polymer diffusion into the solution. All of these are thermally activated processes governed by Arrhenius-type relationships [55]. Consequently, increasing the temperature enhances both solubility and dissolution rate. Regarding the solvent-to-waste ratio, once a sufficient amount of solvent is available to ensure effective polymer–solvent contact, swelling, dissolution, and diffusion, further increases have little additional impact. However, lower ratios are not practically feasible due to the high viscosity of the resulting polymer solution. Therefore, within the solvent-to-waste ratio range investigated in this study, temperature emerges as the dominant factor.

This visualization points out the need to carefully optimize the temperature to effectively control the PP dissolution and minimize *r*-Polymer contamination. The best conditions for minimizing PP concentration, which also coincided with those for maximizing PE, were a temperature of 90 °C, a solvent-to-waste ratio of 30 mL/g, an agitation speed of 400 rpm, and a time of 30 min.

### 3.4. Model Validation and Optimum Conditions Assessment

#### 3.4.1. Validation Experiments

Two validation experiments (VE1 and VE2) were conducted within the range of operating conditions used to develop the models (see Table 1) with the objective of assessing the model’s accuracy in predicting the total yield and the PE content. The results, along with their relative deviation (ε), are presented in Table 5. Further details of the experiments, including operating conditions and response results of replicates, are available in Table S7 in the Supplementary Material.

The operating conditions of VE1 were 95 °C, dissolution time of 30 min, solvent-to-waste ratio of 15 mL/g, and agitation speed of 400 rpm, for which the experimental total yield ($\eta_{\mathrm{Total}}^{\exp}$) was 37.0% and the model prediction was $\eta_{\mathrm{Total}}^{\mathrm{pred}}=\;34.5$%,i.e., the relative deviation is $\varepsilon_{\eta_{\mathrm{Total}}}=\;6.8$%. Regarding PE content, a common value of 99.2 wt% was obtained for both experimental and prediction. For VE2, the operating conditions were T=105 °C, t=30 min, S/W= 15 mL/g, and A=400 rpm, for which $\eta_{\mathrm{Total}}^{\exp}$= 42.5% and $w_{\mathrm{PE}}^{\exp}=\;77.8$%. The modeling predictions resulted in deviations of −13.4% and −12.3%, respectively.

For VE1, the predictions were accurate with minimal deviation. However, in VE2, the increased PP content likely complicated quantification by DSC, as overlapping melting peaks made it more difficult to accurately quantify the individual polymers. The deviations between experimental and predicted values remained within the generally accepted 15% deviation for experimental validation of complex processes [56].

#### 3.4.2. Optimum Conditions Assessment

In the previous sections, the statistical analysis and modeling of $\eta_{\mathrm{Total}}$, $w_{\mathrm{PE}}$, and $w_{\mathrm{PP}}$ were performed and validated. The maximum $\eta_{\mathrm{Total}}$ was achieved at 110 °C, 30 min, 100 rpm, and 30 mL/g, while the maximum PE and minimum PP contents were achieved at 90 °C, 30 min, 400 rpm, and 30 mL/g. The conditions favoring high yield had the opposite impact on the PE and PP desired outcomes.

For the process under study, the optimization focused only on the $\eta_{\mathrm{Total}}$ and $w_{\mathrm{PE}}$ models, since the maximization of $w_{\mathrm{PE}}$ simultaneously imposes the minimization of $w_{\mathrm{PP}}$. Furthermore, it is advantageous to use $w_{\mathrm{PE}}$ instead of $w_{\mathrm{PP}}$, as the former model exhibits better performance (compare the data in Table 3 and Table 4).

The primary optimization goal was to achieve a PE content of at least 95 wt% (i.e., a PP contamination of at most 5 wt%) to ensure compatibility between PE and PP. This threshold is crucial for preserving the mechanical properties and improving economic viability of the recycled polymer, avoiding the need for compatibilizers [38,39]. The optimization aimed to identify the least severe conditions that met this criterion while maximizing the yield, and the theoretical optimal operating conditions were: 100 °C, 30 min, 15 mL/g, and 400 rpm, resulting in $\eta_{\mathrm{Total}}=40.5$% and $w_{\mathrm{PE}}=98.3$ wt%. Experimental results were $\eta_{\mathrm{Total}}=39.1$% and $w_{\mathrm{PE}}=97.7$ wt%, with corresponding relative deviations of −3.6% and −0.61%, respectively (see Table 5). It is worth noting that this optimum assessment can also be taken as an additional successful validation of the various models; hence, both are reported in Table 5.

**Figure 11:**
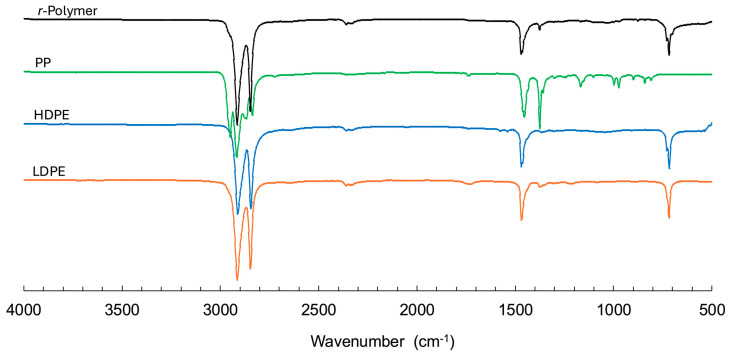
FTIR spectra of *r*-Polymer obtained at optimal operating conditions compared to virgin polymers (LDPE, HDPE, PP).

**Figure 12:**
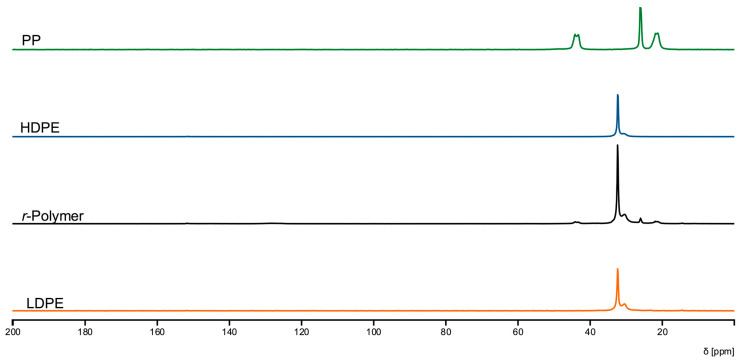
Solid-state NMR spectra of *r*-Polymer obtained at optimal operating conditions compared to virgin polymers (LDPE, HDPE, PP).

The chemical and structural characteristics of the *r*-Polymer obtained under optimized conditions were further examined by FTIR (Figure 11), solid-state ^13^C NMR (Figure 12), and XRD (Figure S4 in the Supplementary Material), and compared with virgin LDPE, HDPE, and PP. All techniques consistently indicate that the recovered material is predominantly PE, with minimal PP contamination. The FTIR spectrum of the *r*-Polymer displays C–H stretching bands in the 2950–2850 cm^−1^ region and C–H bending vibrations near 1470 cm^−1^ and 720 cm^−1^, typical of PE. The solid-state ^13^C NMR spectrum of the *r*-Polymer is dominated by the methylene (–CH_2_–) resonance at 32 ppm, characteristic of PE. Minor signals approximately 22 ppm, 26 ppm and 44 ppm are consistent with methyl and methylene environments associated with PP, confirming the presence of PP only at low concentrations. No additional signals associated with oxygen-containing species were detected by FTIR and NMR within the sensitivity limits of the techniques. XRD analysis further supports these findings, showing diffraction patterns characteristic of semicrystalline PE and PP.

Furthermore, under the optimum conditions, 60.1% of MPPW remained as an undissolved residue. This residue was a complex mixture of polymers originally present in the MPPW, with a higher content of PET, along with remaining undissolved polyolefins. Upon PE recovery, the undissolved residue can be managed through other recycling strategies, such as depolymerization of the PET fraction, as explored elsewhere [40], or by current incineration and landfill disposal. 

## 4. Conclusions

This study systematically optimized key operating conditions for the dissolution–precipitation of MPPW to recover PE. Using a DoE approach and RSM with a Box–Behnken design, critical parameters, including temperature (90–110 °C), dissolution time (30–60 min), solvent-to-waste ratio (15–30 mL/g), and agitation (400–1000 rpm), were analyzed. The goal was to maximize the total yield and PE composition while ensuring PP content below 5 wt% to guaranty combability between PP and PE in the recovered polymer, avoiding the need for compatibilizers.

Dissolution temperature was identified as the most critical factor in the dissolution–precipitation process, with higher temperatures enhancing polymer solubility and mass transfer, thereby increasing overall dissolution yield and kinetics. However, higher temperatures also increase PP selectivity, which leads to lower PE purity. As a result, a careful balance was required to achieve the highest possible yield while maintaining the desired PE purity. Additionally, factors such as dissolution time, agitation speed, and solvent-to-waste ratio were important for improving the mass transfer and the final dissolution yield. Although their individual impacts were less notable, they resulted in synergistic effects when combined.

The statistical analysis and modeling validated temperature as the most significant factor for both yield and composition of the *r*-Polymer, with higher temperatures consistently leading to higher yields and higher PP content, requiring a trade-off to achieve optimal process conditions. The study identified the optimal conditions as 100 °C, 30 min, 400 rpm, and 15 mL/g of toluene-to-MPPW ratio, achieving a total yield of 39.1% and a PE composition of 97.7 wt%.

This study highlights the importance of optimizing MPPW recycling processes to improve resource efficiency and support sustainability goals. By achieving high PE recovery with minimal PP contamination, the optimized process enhances the quality and potential applications of the recovered material while reducing dependence on virgin polymers, in line with circular economy principles. Future research should focus on validating the processability and mechanical performance of the recovered material for use in new products within both closed- and open-loop recycling systems. Additional work is also needed to refine the process, including exploring alternative solvent/polymer separation methods beyond high-speed centrifugation, which can be costly at an industrial scale, improving solvent recovery, scaling up to more realistic processing units, and assessing the economic and environmental feasibility of the upscaled process.

## Figures and Tables

**Table 1:** Coded and actual levels of the experimental factors.

Factor	Level Correspondence		
	Low (−1)	Medium (0)	High (+1)
Dissolution temperature (T, °C)	90	100	110
Dissolution time (t, min)	30	45	60
Solvent-to-waste ratio ((S/W), mL/g)	15	22.5	30
Agitation speed (A, rpm)	400	700	1000

**Table 2:** Regression coefficients for the coded full model of the total dissolution yield and corresponding statistical analysis.

Coefficient	Variable	$\eta_{\mathrm{Total}}$ (%)				
		Coefficient	*p*-Value	$R^{2}$	$R_{\mathrm{adj}}^{2}$	AARD (%)
$b_{0}$	Constant	30.650	0.000	0.959	0.943	5.52
$b_{1}$	T	16.120	0.000			
$b_{11}$	$T^{2}$	4.900	0.000			
$b_{2}$	t	0.968	0.107			
$b_{22}$	$t^{2}$	6.549	0.000			
$b_{3}$	S/W	1.170	0.053			
$b_{33}$	${\left(S/W\right)}^{2}$	−0.817	0.019			
$b_{4}$	A	−0.817	0.171			
$b_{44}$	$A^{2}$	2.105	0.897			
$b_{12}$	T×t	−1.739	0.095			
$b_{13}$	$T\times \left(S/W\right)$	2.453	0.020			
$b_{14}$	T×A	−0.621	0.544			
$b_{23}$	$t\times \left(S/W\right)$	1.790	0.086			
$b_{24}$	t×A	−2.155	0.041			
$b_{34}$	$\left(S/W\right)\times A$	2.219	0.035			

**Table 3:** Statistical analysis of full and reduced models for the PE content ($w_{\mathrm{PE}}$) in *r*-Polymer.

Model	Number of Terms	$R^{2}$	$R_{\mathrm{adj}}^{2}$	AARD (%)
Full (Equation (5))	15	0.974	0.964	1.69
Reduced (Equation (9))	4	0.967	0.965	1.62

**Table 4:** Statistical analysis of full and reduced models for the PP relative content in *r*-Polymer.

Model	Number of Terms	$R^{2}$	$R_{\mathrm{adj}}^{2}$	AARD (%)
Full (Equation (5))	15	0.974	0.964	44.7
Reduced (Equation (10))	4	0.967	0.965	46.1

**Table 5:** Validation experiments (VE1 and VE2) results for total yield and PE content. The OC row corresponds to the assessment of the optimum conditions. $\varepsilon_{\eta_{\mathrm{Total}}}$ and $\varepsilon_{w_{\mathrm{PE}}}$ are the deviations between experimental and prediction values.

Exp.	Operating Conditions				$\eta_{\mathrm{Total}}$			$w_{\mathrm{PE}}$		
	T (°C)	t (min)	S/W (mL/g)	A(rpm)	$\eta_{\mathrm{Total}}^{\exp}$(%)	$\eta_{\mathrm{Total}}^{\mathrm{pred}}$(%)	$\varepsilon_{\eta_{\mathrm{Total}}}$(%)	$w_{\mathrm{PE}}^{\exp}$(%)	$w_{\mathrm{PE}}^{\mathrm{pred}}$(%)	$\varepsilon_{w_{\mathrm{PE}}}$(%)
VE1	95	30	15	400	37.0	34.5	6.8	99.2	99.2	0
VE2	105	30	15	400	42.5	48.2	−13.4	77.8	87.4	−12.3
OC	100	30	15	400	39.1	40.5	−3.6	97.7	98.3	−0.61

## Data Availability

The original contributions presented in this study are included in the article/Supplementary Material. Further inquiries can be directed to the corresponding author.

## Supplementary Materials

The following supporting information can be downloaded at: https://www.mdpi.com/article/10.3390/polym18050638/s1, Table S1. Composition of MPPW determined by selective dissolution–precipitation; Table S2. Summary of the results obtained from the dissolution–precipitation of virgin polymers in toluene, xylene, tetrahydrofuran, dimethyl sulfoxide, and N-methylpyrrolidone compared with predictions based on Hildebrand and Hansen solubility parameters; Table S3. List of experiments conducted for the DoE featuring the operating conditions and response results. The DoE included four factors and three levels, one central point, one replicate, and follows the Box–Behnken design structure; Table S4. Parameters of the full and reduced models for the total yield response; Table S5. Parameters of the full and reduced models for the PE content in the *r*-Polymer; Table S6. Parameters of the full and reduced models for the PP content in the *r*-Polymer; Table S7. Validation experiments, including replicates, operating conditions, and response results. VEiR stands for validation of experiment i replicate, and OC refers to optimum conditions; Figure S1. Response surfaces obtained for $\eta_{\mathrm{Total}}$ as a function of temperature and (a) agitation speed at fixed time (45 min) and solvent-to-waste ratio (22.5 mL/g), and (b) solvent-to-waste ratio at fixed time (45 min) and agitation (700 rpm). Black dots represent experimental data, and $\left(\eta_{\mathrm{Total}}\left(T,\;A\right)\right)$ as well as $\left(\eta_{\mathrm{Total}}\;\left(T,(S/W\right))\right)$ are given by Equation (8) (reduced model); Figure S2. Pareto chart of the relative content of PE ($w_{\mathrm{PE}},\;\mathrm{wt}\%)$ response according to different operating variables. Dark bars denote positive contribution (desired effect), and light bars denote negative contribution (undesired effect). The red vertical line corresponds to the significance level at 95% confidence interval; Figure S3. Pareto chart of the relative content of PP ($w_{\mathrm{PP}},\;\mathrm{wt}\%)$ response according to different operating variables. Dark bars denote positive contribution (desired effect) and light bars denote negative contribution (undesired effect). The red vertical line corresponds to the significance level at 95% confidence interval; Figure S4. XRD diffractograms of *r*-Polymer obtained at optimal operating conditions compared to virgin polymers (LDPE, HDPE, PP).

## Abbreviations

The following abbreviations are used in this manuscript:

A	Agitation speed
AARD	Average absolute relative deviation
BB	Box–Behnken
DMSO	Dimethyl sulfoxide
DoEs	Design of experiments
DSC	Differential scanning calorimetry
E	Melting energy
EDS	Energy dispersive X-ray spectroscopy
EVA	Poly(ethylene-*co*-vinyl acetate)
EVOH	Poly(ethylene-*co*-vinyl alcohol)
FTIR	Fourier transform infrared spectroscopy
HDPE	High-density polyethylene
LDPE	Low-density polyethylene
m	Mass
MPP	Multilayer plastic packaging
MPPW	Multilayer plastic packaging waste
NMP	N-methylpyrrolidone
PA	Polyamide
PE	Polyethylene
PE-g-MA	Polyethylene grafted with maleic anhydride
PET	Poly(ethylene terephthalate)
PO	Polyolefin
PP	Polypropylene
PS	Polystyrene
$R^{2}$	Coefficient of determination
$R_{\mathrm{adj}}^{2}$	Adjusted coefficient of determination
*r*-Polymer	Recovered polymer
RSM	Response surface methodology
STRAP	Solvent-targeted recovery and precipitation
t	Dissolution time
T	Dissolution temperature
$T_{m}$	Melting temperature
TGA	Thermogravimetric analysis
THF	Tetrahydrofuran
w	Relative composition
w′	Mass fraction
Greek letters	
α	Heating rate
β	Coded regression coefficient
$\Delta H_{m}$	Melting enthalpy
ε	Relative deviation
η	Dissolution yield
$\chi_{12}$	Flory–Huggins interaction parameter
Subscripts	
exp.	Relative to experimental value
pred.	Relative to predicted value
ref.	Relative to reference value
Total	Relative to the total dissolved material
