# Supplementary material for: Polyethylene Recovery from Multilayer Plastic Packaging Waste
Source: Polymers (Basel). 2026 Mar 5;18(5):638. doi: 10.3390/polym18050638 (PMC12986593; doi:10.3390/polym18050638)
Supplement: Supplementary file 1 [file polymers-18-00638-s001.zip › polymers-4143164-supplementary.pdf]

Supplementary Material

Polyethylene Recovery from Multilayer Plastic Packaging Waste

Anareth Cavuquila [1,†], Luanna Maia [1,2,3,†], Germano A. Carreira [4], Inês Portugal [1], Carlos M. Silva [3,*] and Ana Barros-Timmons [1]

1 CICECO—Aveiro Institute of Materials, Department of Chemistry, University of Aveiro, 3810-193 Aveiro, Portugal

2 LEPABE, ALiCE, Faculty of Engineering, University of Porto, Rua Dr. Roberto Frias, 4200-465 Porto, Portugal

3 CERES, Department of Chemical Engineering, Faculty of Sciences and Technology, University of Coimbra, Rua Sílvio Lima, Polo II, 3030-790, Coimbra, Portugal

4 i9Green, Av. D. José Alves Correia da Silva, Edifício Vela Sul 2º D, Rotunda Sul, Cova da Iria, 2495-402 Fátima, Portugal

* Correspondence: carlos.manuel@ua.pt

† These authors contributed equally to this work.

Contents

Tables

Table S1. Composition of MPPW determined by selective dissolution–precipitation. 3

Table S2. Summary of the results obtained from the dissolution–precipitation of virgin polymers in toluene, xylene, tetrahydrofuran, dimethyl sulfoxide, and N-methylpyrrolidone compared with predictions based on Hildebrand and Hansen solubility parameters. 4

Table S3. List of experiments conducted for the DoE featuring the operating conditions and response results. The DoE included four factors and three levels, one central point, one replicate, and follows the Box–Behnken design structure. 5

Table S4. Parameters of the full and reduced models for the total yield response. 8

Table S5. Parameters of the full and reduced models for the PE content in the *r*-Polymer.... 10

Table S6. Parameters of the full and reduced models for the PP content in the *r*-Polymer.... 12

Table S7. Validation experiments, including replicates, operating conditions, and response results. VEiR stands for validation of experiment i replicate, and OC refers to optimum conditions. 13

Figures

Figure S1. Response surfaces obtained for η_{Total} as a function of temperature and (a) agitation speed at fixed time (45 min) and solvent-to-waste ratio (22.5 mL/g), and (b) solvent-to-waste ratio at fixed time (45 min) and agitation (700 rpm). Black dots represent experimental data, and $\eta_{\text{Total}}(T, A)$ as well as $\eta_{\text{Total}}(T, \left(\frac{S}{W}\right))$ are given by Equation (8) (reduced model). 9

Figure S2. Pareto chart of the relative content of PE (w_{PE}, wt%) response according to different operating variables. Dark bars denote positive contribution (desired effect), and light bars denote negative contribution (undesired effect). The red vertical line corresponds to the significance level at 95% confidence interval. 9

Figure S3. Pareto chart of the relative content of PP (w_{PP}, wt%) response according to different operating variables. Dark bars denote positive contribution (desired effect) and light bars denote negative contribution (undesired effect). The red vertical line corresponds to the significance level at 95 % confidence interval. 11

Figure S4. XRD diffractograms of *r*-Polymer obtained at optimal operating conditions compared to virgin polymers (LDPE, HDPE, PP)..13

Table S1. Composition of MPPW determined by selective dissolution–precipitation.

Dissolution step	Solvent	Temperature (ºC)	Yield (wt%)	Composition (FTIR)
1	Toluene	24	6.0	PS, Additives
2	Toluene	65	2.3	PS, Additives
3	Toluene	72	12.0	LDPE-rich
4	Toluene	100	23.0	HDPE-rich
5	Toluene	110	14.8	HDPE and PP-rich
6	NMP	132	20.5	PET and PA-rich

Table S2. Summary of the results obtained from the dissolution–precipitation of virgin polymers in toluene, xylene, tetrahydrofuran, dimethyl sulfoxide, and N-methylpyrrolidone compared with predictions based on Hildebrand and Hansen solubility parameters.

Polymer	Form	Hildebrand model prediction	Hansen model prediction	Experimental observation	Dissolution temperature (°C)	Precipitation temperature (°C)
Dissolution in toluene						
LDPE	Granular	D	D	D	72	68
HDPE	Granular	D	D	D	100	94
PP	Pellet	D	D	D	110	80
PET	Pellet	ND	ND	ND	-	-
PS	Pellet	D	D	D	24	NP
EVA	Pellet	D	D	D	72	61
PA	Film	ND	ND	ND	-	-
Dissolution in xylene						
LDPE	Granular	D	D	D	72	65
HDPE	Granular	D	D	D	100	96
PP	Pellet	D	D	D	105	88
PET	Pellet	ND	ND	ND	-	-
PS	Pellet	D	D	D	24	NP
EVA	Pellet	D	D	D	95	86
PA	Film	ND	ND	ND	-	-
Dissolution in tetrahydrofuran						
LDPE	Granular	D	D	D	74	68
HDPE	Granular	D	ND	ND	-	-
PP	Pellet	D	ND	ND	-	-
PET	Pellet	ND	D	ND	-	-
PS	Pellet	D	D	D	24	NP
EVA	Pellet	D	D	D	65	60
PA	Film	ND	D	ND	-	-
Dissolution in N-methylpyrrolidone						
LDPE	Granular	ND	ND	ND	-	-
HDPE	Granular	ND	ND	ND	-	-
PP	Pellet	ND	ND	ND	-	-
PET	Pellet	D	D	D	132	-
PS	Pellet	ND	ND	D	100	NP
EVA	Pellet	ND	ND	ND	-	-
PA	Film	D	ND	D	172	98
Dissolution in dimethyl sulfoxide						
LDPE	Granular	ND	ND	ND	-	-
HDPE	Granular	ND	ND	ND	-	-
PP	Pellet	ND	ND	ND	-	-
PET	Pellet	ND	ND	D	157	NP
PS	Pellet	ND	ND	D	174	140
EVA	Pellet	ND	ND	ND	-	-
PA	Film	ND	ND	D	157	90

D – dissolved, ND – not dissolved, ND – not precipitated

Table S3. List of experiments conducted for the DoE featuring the operating conditions and response results. The DoE included four factors and three levels, one central point, one replicate, and follows the Box–Behnken design structure.

t (min)	S/W (mL/g)	A (rpm)	T (° C)	η_{Total} (%)		w_{PE} (wt%)		w_{PP} (wt%)	
				Exp.	Average	Exp.	Average	Exp.	Average
60	30	700	100	46.83	44.99	98.83	98.83	1.17	1.17
60	30	700	100	43.15		98.84		1.16	
45	22.5	400	110	57.85	57.77	74.82	74.69	25.18	25.31
45	22.5	400	110	57.69		74.56		25.44	
45	30	700	110	59.74	57.64	75.74	72.91	24.26	27.09
45	30	700	110	55.54		70.08		29.92	
30	22.5	700	110	54.12	55.29	73.32	75.43	26.68	24.57
30	22.5	700	110	56.46		77.53		22.47	
30	22.5	1000	100	44.48	41.52	97.43	97.95	2.57	2.05
30	22.5	1000	100	38.56		98.48		1.52	
30	15	700	100	38.82	40.62	96.78	97.77	3.22	2.23
30	15	700	100	42.41		98.75		1.25	
30	22.5	700	90	21.05	21.02	100.00	100.00	0.00	0.00
30	22.5	700	90	20.99		100.00		0.00	
45	15	400	100	38.85	36.90	94.48	95.68	5.52	4.32
45	15	400	100	34.95		96.87		3.13	
60	22.5	700	110	55.09	57.12	75.46	72.68	24.54	27.32
60	22.5	700	110	59.16		69.90		30.10	

45	15	700	110	51.66	52.97	81.69	79.29	18.31	20.71
45	15	700	110	54.28		76.89		23.11	
60	22.5	1000	100	38.36	39.12	99.72	99.65	0.28	0.35
60	22.5	1000	100	39.87		99.59		0.41	
45	30	1000	100	37.73	36.39	98.80	98.63	1.20	1.37
45	30	1000	100	35.06		98.47		1.53	
45	30	400	100	32.93	33.94	99.48	99.04	0.52	0.96
45	30	400	100	34.94		98.60		1.40	
60	22.5	400	100	41.16	42.25	98.90	98.97	1.10	1.03
60	22.5	400	100	43.33		99.03		0.97	
45	22.5	400	90	26.39	23.35	100.00	100.00	0.00	0.00
45	22.5	400	90	20.31		100.00		0.00	
45	30	700	90	20.79	19.99	100.00	100.00	0.00	0.00
45	30	700	90	19.19		100.00		0.00	
45	22.5	1000	90	19.53	20.48	100.00	100.00	0.00	0.00
45	22.5	1000	90	21.44		100.00		0.00	
45	22.5	1000	110	51.68	52.42	70.86	69.19	29.14	30.81
45	22.5	1000	110	53.16		67.52		32.48	
30	30	700	100	41.75	42.82	98.97	99.31	1.03	0.69
30	30	700	100	43.89		99.65		0.35	
60	22.5	700	90	31.21	29.81	100.00	100.00	0.00	0.00
60	22.5	700	90	28.41		100.00		0.00	

30	22.5	400	100	37.62	36.03	98.51	97.79	1.49	2.21
30	22.5	400	100	34.44		97.07		2.93	
45	15	700	90	23.64	25.13	100.00	100.00	0.00	0.00
45	15	700	90	26.63		100.00		0.00	
60	15	700	100	33.61	35.62	97.00	97.45	3.00	2.55
60	15	700	100	37.64		97.90		2.10	
45	22.5	700	100	28.33	30.65	98.58	97.52	1.42	2.48
45	22.5	700	100	32.96		96.45		3.55	
45	15	1000	100	30.77	30.49	99.60	99.80	0.40	0.20
45	15	1000	100	30.20		100		0.40	

Table S4. Parameters of the full and reduced models for the total yield response.

Model	Effect summary		Statistical parameters				
	Effect	p-value	p-value (ANOVA)	R^2	R^2_{adj}	AARD (%)	Estimate
Full	Constant	0.000	< 0.0001	0.959	0.943	5.52	30.650
	T	0.000					16.120
	t^2	0.000					6.549
	T^2	0.000					4.900
	$(S/W)^2$	0.019					-0.818
	$T\times(S/W)$	0.021					2.453
	$A\times(S/W)$	0.035					2.219
	$t\times A$	0.041					-2.155
	S/W	0.053					1.170
	$t\times(S/W)$	0.086					1.790
	A^2	0.897					2.105
	$T\times t$	0.095					-1.739
	t	0.107					0.968
	A	0.171					-0.818
	$T\times A$	0.544					-0.621
Reduced	Constant	0.000	< 0.0001	0.938	0.928	6.94	33.610
	T	0.000					16.120
	t^2	0.000					5.187
	T^2	0.001					3.537
	$T\times(S/W)$	0.029					2.454
	$A\times(S/W)$	0.047					2.219
	$t\times A$	0.054					-2.155
	$(S/W)^2$	0.111					1.600

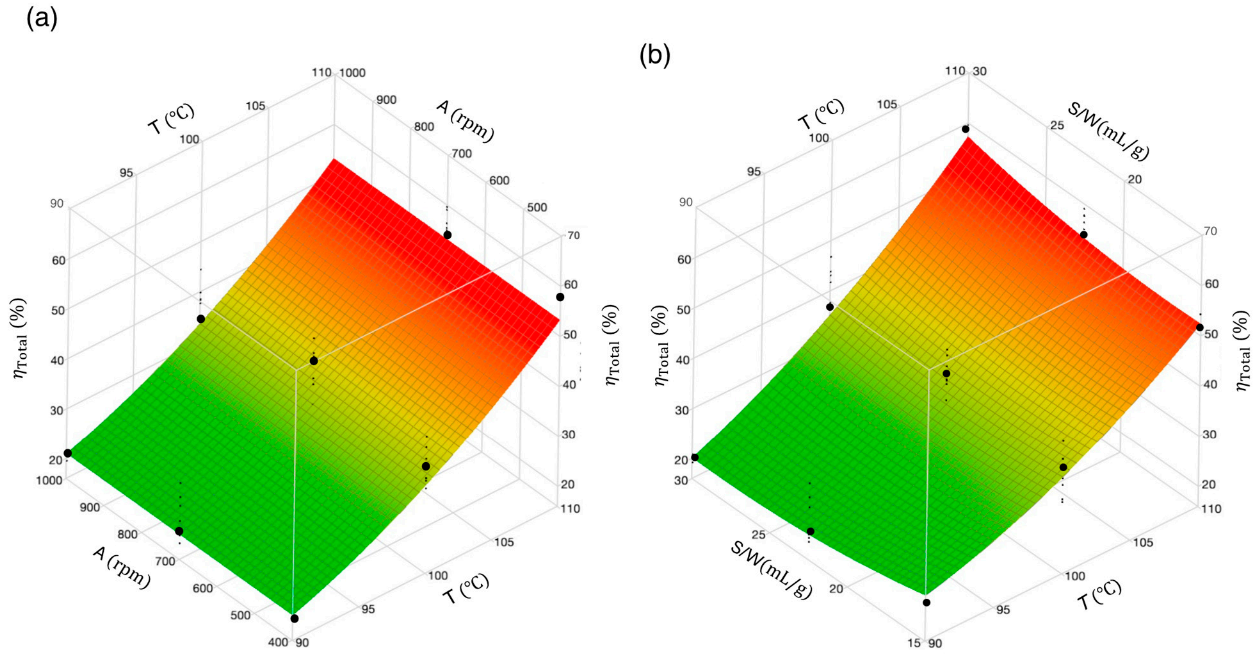

Figure S1. Response surfaces obtained for η_{Total} as a function of temperature and (a) agitation speed at fixed time (45 min) and solvent-to-waste ratio (22.5 mL/g), and (b) solvent-to-waste ratio at fixed time (45 min) and agitation (700 rpm). Black dots represent experimental data, and $\left(\eta_{\text{Total}}(T,A)\right)$ as well as $\left(\eta_{\text{Total}}\,(T,(S/W))\right)$ are given by Equation (8) (reduced model).

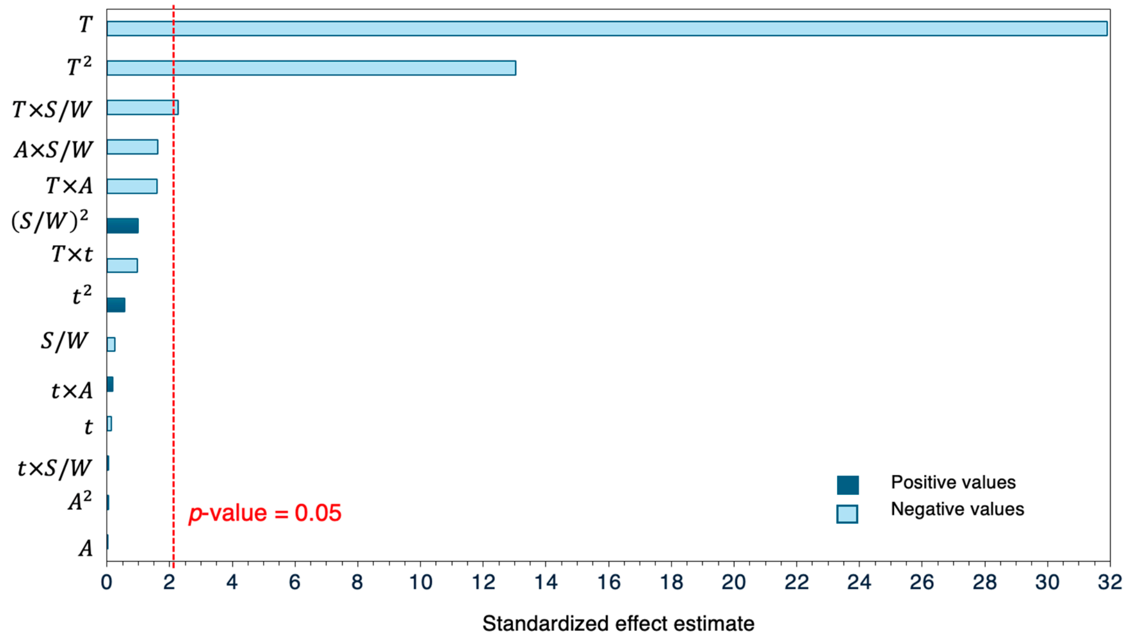

Figure S2. Pareto chart of the relative content of PE (w_{PE}, wt%) response according to different operating variables. Dark bars denote positive contribution (desired effect), and light

bars denote negative contribution (undesired effect). The red vertical line corresponds to the significance level at 95% confidence interval.

Table S5. Parameters of the full and reduced models for the PE content in the *r*-Polymer.

Model	Effect summary		Statistical parameters				
	Effect	p-value	p-value (ANOVA)	R^2	R^2_{adj}	AARD (%)	Estimate
Full	Constant	0.000	< 0.0001	0.974	0.964	1.69	97.730
	T	0.000					-12.120
	T^2	0.000					-10.250
	$T \times (S/W)$	0.038					-1.516
	$T \times A$	0.071					-1.320
	$A \times (S/W)$	0.134					-1.035
	$(S/W)^2$	0.329					0.815
	$T \times t$	0.359					-0.656
	t^2	0.565					0.468
	S/W	0.809					-0.114
	A	0.858					-0.0917
	$t \times A$	0.860					0.120
	t	0.898					-0.060
	$t \times (S/W)$	0.957					-0.036
	A^2	0.960					-0.062
Reduced	Constant	0.000	< 0.0001	0.967	0.965	1.62	98.330
	T	0.000					-12.120
	T^2	0.000					-10.600
	$T \times (S/W)$	0.032					-1.516

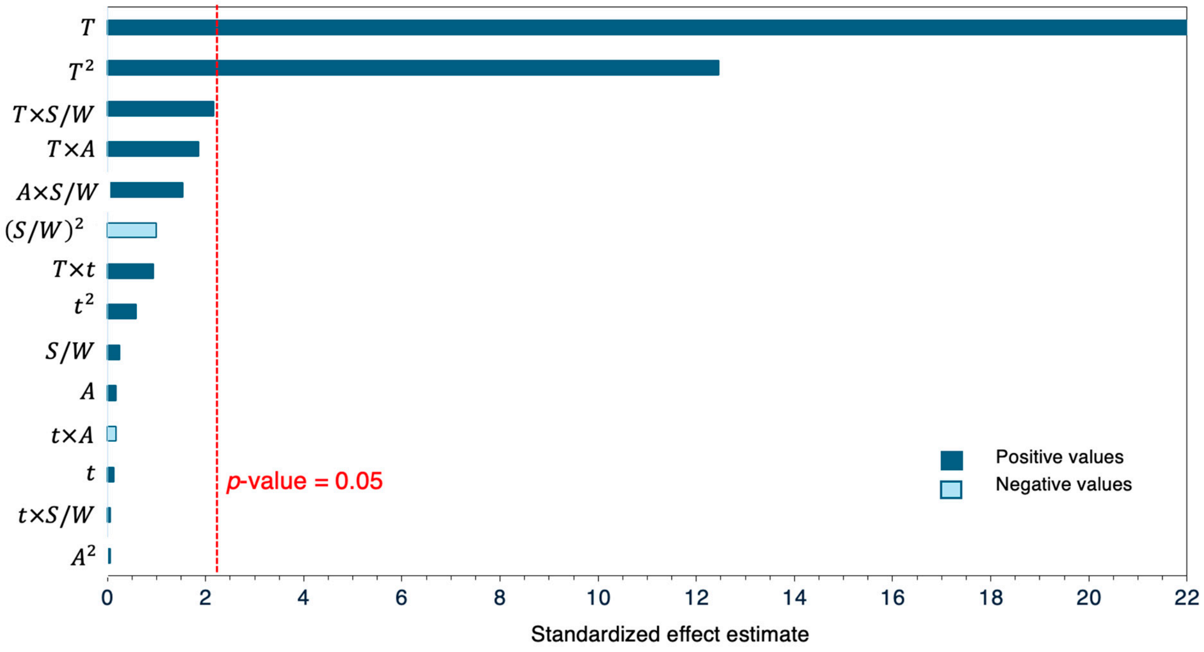

Figure S3. Pareto chart of the relative content of PP (w_{PP}, wt%) response according to different operating variables. Dark bars denote positive contribution (desired effect) and light bars denote negative contribution (undesired effect). The red vertical line corresponds to the significance level at 95 % confidence interval.

Table S6. Parameters of the full and reduced models for the PP content in the *r*-Polymer.

Model	Effect summary		Statistical parameters				
	Source	p-value	p-value (ANOVA)	R^2	R^2_{adj}	AARD (%)	Estimate
Full	Constant	0.000	< 0.0001	0.974	0.965	44.7	2.270
	T	0.000					12.120
	T^2	0.000					10.250
	$T \times (S/W)$	0.038					1.516
	$T \times A$	0.071					1.320
	$A \times S/W$	0.134					1.035
	$(S/W)^2$	0.329					-0.815
	$T \times t$	0.359					0.656
	t^2	0.565					-0.468
	S/W	0.808					0.114
	A	0.858					0.092
	$t \times A$	0.860					-0.120
	t	0.898					0.060
	$t \times (S/W)$	0.957					0.036
	A^2	0.960					0.062
Reduced	Constant	0.000	< 0.0001	0.967	0.965	46.1	1.660
	T	0.000					12.120
	T^2	0.000					10.600
	$T \times (S/W)$	0.032					1.516

Table S7. Validation experiments, including replicates, operating conditions, and response results. VEiR stands for validation of experiment i replicate, and OC refers to optimum conditions.

Exp.	t (min)	S/W (mL/g)	A (rpm)	T (° C)	η_{Total} (%)		w_{PE} (wt%)		w_{PP} (wt%)	
					Exp.	Average	Exp.	Average	Exp.	Average
VE1	30	15	400	95	40.31	37.04	99.02	99.18	0.98	0.83
VE1R	30	15	400	95	33.77		99.33		0.67	
VE2	30	15	400	105	39.56	42.54	75.05	77.83	24.95	22.17
VE2R	30	15	400	105	45.52		80.60		19.40	
OC1	100	30	15	400	39.21	39.10	98.73	97.70	1.27	2.30
OC2	100	30	15	400	38.91		96.66		3.34	

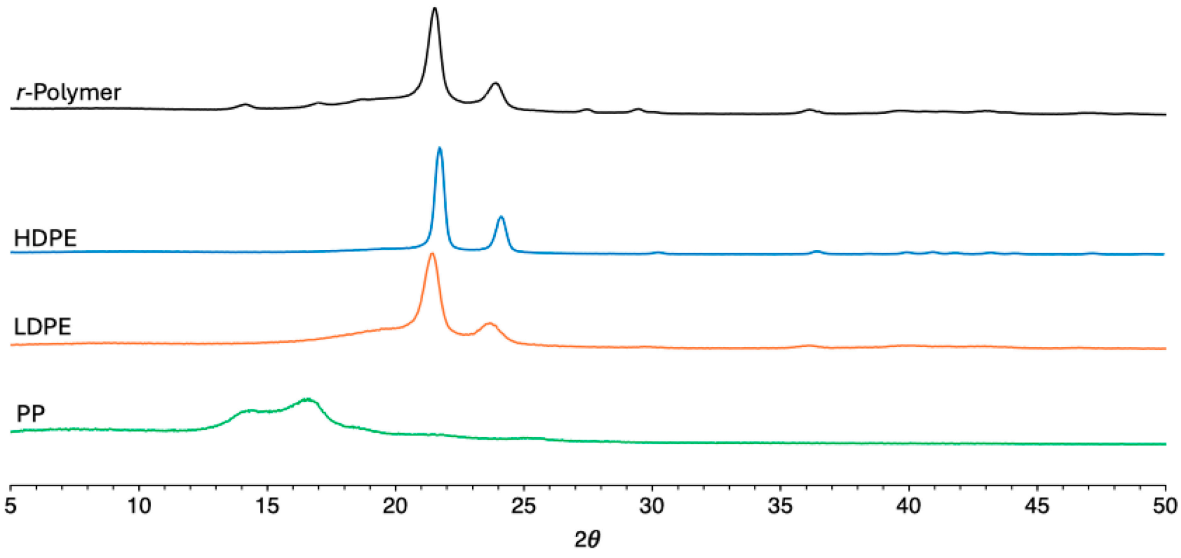

Figure S4. XRD diffractograms of *r*-Polymer obtained at optimal operating conditions compared to virgin polymers (LDPE, HDPE, PP).
